# The Effect of Disinfectants on Quinolone Resistant *E. coli* (QREC) in Biofilm

**DOI:** 10.3390/microorganisms8111831

**Published:** 2020-11-20

**Authors:** Ane Mohr Osland, Lene K. Vestby, Live L. Nesse

**Affiliations:** Norwegian Veterinary Institute, Ullevålsvn 68, P.O. Box 750 Sentrum, 0106 Oslo, Norway; lene.karine.vestby@vetinst.no (L.K.V.); live.nesse@vetinst.no (L.L.N.)

**Keywords:** biofilm, QREC, disinfection, *E. coli*, antimicrobial resistance

## Abstract

The aim of disinfection is to reduce the number of microorganisms on surfaces which is a challenge due to biofilms. In the present study, six quinolone resistant *Escherichia coli* (QREC) strains with three different biofilm matrix compositions were included to assess the log_10_ colony forming units (CFU) reduction effect of three disinfectants at various exposure times on biofilm of different ages and morphotypes. Biofilm was formed on stainless steel coupons for two and five days before transferred to tubes with Virocid 0, 25%, VirkonS 1%, and TP990 1% and left for various exposure times. The biofilms were scraped off and serial dilutions were spread on blood agar plates where colony forming units (CFU) were counted. A mean log_10_ CFU reduction ≥4 was seen on two-day-old biofilm with VirkonS and Virocid (30 min) but not on five-day old biofilm. TP990 did not display sufficient effect under the conditions tested. The bactericidal effect was inferior to that reported on planktonic bacteria. The findings of this study should be considered when establishing both disinfectant routines and standard susceptibility tests, which further should accommodate *E. coli* biofilms and not only *Pseudomonas* as is the case today.

## 1. Introduction

World health organization (WHO) considers antibiotic resistance to be one of the biggest threats to global health, food security, and development today, and it focuses particularly on reducing resistance to antimicrobials defined as critically important for human medicine, e.g., quinolones and fluoroquinolones [1,2]. Bacterial resistance to these compounds has increased yearly from 2013 to 2016 and is continuing to rise in Europe. In Norway, the use of fluoroquinolones in livestock is minute. Nevertheless, the Norwegian monitoring program for antimicrobial resistance (NORM-VET) has, since the implementation of a new selective method in 2014, found a low-level resistance in a high proportion of the samples from the broiler production chain [3]. Seeing that quinolones are not used regularly in this industry, it is postulated that population density and production environment may pose a critical role [4] in the development of resistance. Failure to eradicate resistant bacteria from the food production environment may contribute to persistence and dissemination.

It is known that *E. coli* can form biofilms on assorted surfaces and at temperatures pertaining to normal conditions in the food production industry [5,6,7]. Previous studies on Norwegian QREC strains isolated from poultry production supported this and showed that they in fact were good biofilm formers at room temperature and hence were likely to persist in reservoirs in animal and food production chains [5]. The low nutrient conditions in this milieu are especially favorable for the formation of biofilms which may consequently facilitate the persistence of QREC [8]. Biofilms are communities of microorganisms that adhere together or to a surface and form a self-embedding matrix of extracellular polymeric substance (EPS), composed of polysaccharides, proteins, and nucleic acids [9,10].

Two extracellular matrix components of *E. coli* biofilms differentiate them into different biofilm morphotypes according to their appearance on agar plates with Congo Red and Coomassie brilliant blue dyes (CR plates). The RDAR morphotype (red, dry, and rough) express cellulose and curli fimbriae, PDAR (pink, dry, and rough) express cellulose solely, BDAR (brown, dry, and rough) express curli only, while SAW (smooth and white) indicates the absence of biofilm growth [11]. The RDAR morphotype is the most studied and the most prevalent [5,7]. Previous studies have indicated that the presence of curli and cellulose may influence both biofilm production and the protective properties of the matrix [12,13]. For example, studies done on *Salmonella* species showed that extracellular components, such as curli fibers, were important in colonization of various surfaces for instance plant surfaces, abiotic surfaces, cell aggregation, and in the air liquid interface. The role of cellulose is less clear, but it has been suggested to play a role in biotic surface colonization and give structural stability to the RDAR morphotype [11,12,14]. A synergistic [15] as well as a counteractive [16] role of cellulose in curli-mediated cell adherence and colonization of solid surfaces has been implied.

Disinfectants are widely used as a prevention and control strategy against infections in the general household, hospitals, food production facilities, and on other premises. In spite of their vast use, we do not know much about the mode of action compared to other biocides, such as antibiotics. Disinfectants have a wider specter of activity than antibiotics and may potentially have several targets. Even though the aim of disinfection is to reduce microorganisms on the innate surfaces by ≥log 4, this level of disinfection might not always be reached. One plausible reason for this is the development of biofilms [9,10], which are challenging to eliminate by sanitation procedures as they stick firmly to diverse surfaces and consist of organic materials such as exopolysaccharides and proteins [17]. Biofilms are often found in hard to clean areas of an establishment and are known to develop in cracks, tubes, and other similar niches. In addition, the dormant cells and their matrix makes them more resilient and more tolerant to outside forces, such as disinfectants and antibiotics, compared to their planktonic correlatives [18,19,20,21]. The permeability of the matrix can also be decreased by various factors such as a change in the microenvironment, cell density, and the age of the biofilm. The two latter factors are strongly correlated and are hard to separate as the biofilm matrix becomes thicker and denser as it ages and colony forming units (CFU) increase. In spite of this, it has been indicated that biofilm age plays a more important role than cell density [22] concerning increased tolerance to biocides. Looking at the aforementioned factors together, it is crucial when designing sanitation routines in food production facilities to take into account the formation of biofilms and also to consider the age of the biofilm to be combated.

The aim of this study was to investigate the effect of three disinfectants commonly used in the poultry production industry in Norway on QREC isolates collected from the broiler production chain. We found that the effect of the disinfectants was influenced both by their composition and the exposure time, as well as by the age of the biofilms and the constituents in the biofilm matrices.

## 2. Materials and Methods

### 2.1. Strains

Six *E. coli* strains, collected and confirmed as QREC in the national surveillance program for antimicrobial resistance in the veterinary and food production (NORM-VET) in 2014 [23], were used in this study (Table 1). Their morphotypes and biofilm forming abilities on glass slides had been determined in an earlier study [5]. Their abilities to form biofilm on stainless steel were confirmed before the disinfection experiments started, by using the same assay as described below but without the disinfection step. All strains were stored at −80 °C in Brain Heart Infusion broth (BHI; Difco, BD, Franklin Lakes, NJ, USA) supplemented with 15% glycerol (Merck KGaA, Darmstadt, Germany) and recovered on blood agar (sheep blood) at 37.0 ± 1.0 °C overnight.

### 2.2. Disinfection Assay

Bacterial blood agar cultures were transferred to 5 mL Luria Bertani broth (Merck KGaA, Darmstadt, Germany) and the Optical density (OD_595_) was adjusted to 1 ± 0, 1(Amersham Biosciences, Ultrospec 10, densitometer). Then, 500 μL of each bacterial suspension was added to 10 mL of LB wo/NaCl (Bacto-tryptone 10 g/L, yeast extract 5 g/L) together with an autoclaved stainless-steel coupon of 75 × 24 × 1 mm (Stainless steel AISI304, 2B Olaf Johansens Eftf. A/S, Oslo, Norway) and incubated at 20 °C ± 1 °C for 48 h (two-day-old biofilm) and 120 h (five-day-old biofilm).

The disinfection assay was performed according to Vestby et al. 2010 [24] with minor modifications. After biofilm formation, the coupon was rinsed in 40 mL sterile saline and transferred to a tube with 10 mL of disinfectant (or saline for controls) for the applicable exposure time. As there were some discrepancies between the disinfectant suppliers’ recommended exposure times, we chose to use 30 min for all three disinfectants, and in addition, 10 min for Virkon S and 15 min for TP 990. The coupon was then moved to a tube with 15 mL Dey-Engley Neutralizing broth (Pepton, yeast extract, dextrose. Difco, BD, NJ, USA) before it was rinsed again and added to a tube containing 20–30 autoclaved glass beads (3 mm, Assistant, Glaswarenfabrik Karl Hecht GmbH & Co KG, Bavaria, Germany) and 5 mL saline. Here, visible biofilm was scraped off both sides of the coupon by using an 18 cm long cell scraper with a blade of 1.8 cm (BD Falcon, Bedford, MA, USA). The coupon was discarded before the tube was vortexed for one minute. An aliquot of 200 μL from each tube was added in triplicates to wells in a microtiter plate (Nunc A/S, Roskilde, Denmark). Serial dilutions were made before plating 100 μL on blood agar (5% sheep blood) and incubation at 37 °C for 24 h. After incubation, the number of CFU of each strain were counted. All experiments were performed at least 3 times. The results were calculated as mean log_10_ CFU reductions, i.e., mean log_10_ CFU of control biofilm—mean log_10_ CFU of treated biofilm [25]. Information on the disinfectants used is given in Table 2.

### 2.3. Statistics

All statistical analyses were performed using Excel vs. 2016 (Microsoft, Redmond, WA, USA). To evaluate the effect of disinfectant treatment, 95% confidence interval (CI95%) of mean reduction was calculated. If the mean ± CI95% did not include 0, the reduction was considered statistically significant. Efficient disinfection effect was defined as a log_10_ CFU reduction ≥4 according to the requirements in the European surface test (2015) [25]. We considered this requirement met when the reduction was not significantly different from 4 or higher, i.e., when the mean reduction ± CI95% included log_10_ CFU ≥ 4. A two-tailed Student’s T-test, with the level of significance set to be *p* ≤ 0.05, was used when comparing disinfectant efficacies on biofilms with different morphology and of different ages.

## 3. Results

**Figure 1:**
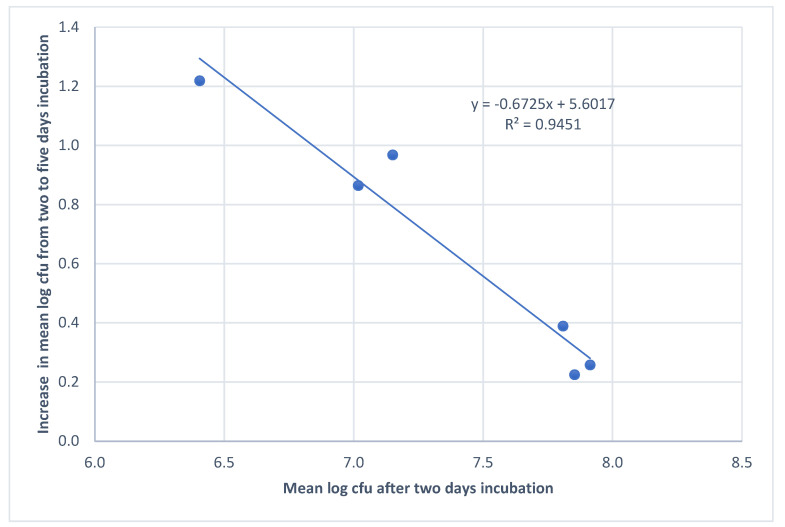
Correlation between mean log_10_ CFU in the biofilm after two days incubation and the increase in mean log_10_ CFU from two to five days.

In this study, we found statistically significantly more CFU in the five-day-old biofilm controls compared to the two-day old (mean log_10_ 8.01 and mean log_10_ 7.35, *p* ≤ 0.05). The strains that had the lowest biofilm production after two days were the ones with the greatest increase toward day five (Table 1, Figure 1).

**Figure 2:**
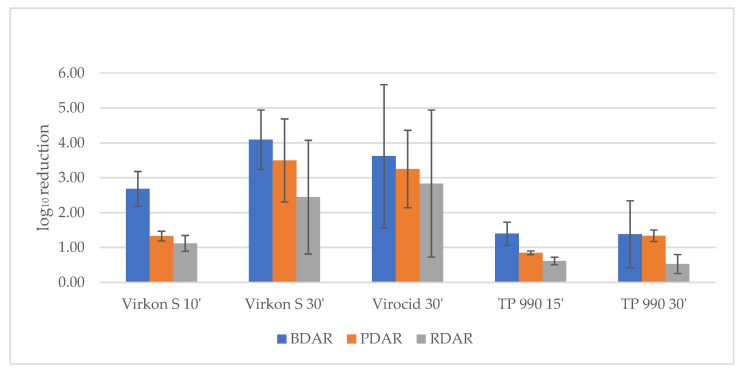
Morphological variation in disinfectant tolerance. The effect on RDAR, BDAR, and PDAR biofilm formed for two days and treated with Virkon S for 10 and 30 min, Virocid for 30 min, and TP990 for 15 and 30 min. Bars indicate standard deviation. ′ = Minutes of disinfectant exposure.

All disinfectant agents used, under the conditions tested in this study, gave a statistically significant decrease in mean log_10_ CFU recovered from both two and five-day old biofilm and at all exposure times (Table 2, Figure 2). However, only Virocid and Virkon S, with an exposure time of 30 min, displayed a mean log_10_ reduction ± CI95% including 4 and could therefore be categorized as efficient on two-day-old biofilms following the requirements described in NS-EN 13697:2015 [25]. Reducing exposure time for Virkon S from 30 to 10 min reduced the reduction of CFU, whereas a reduction from 30 to 15 min for TP 990 had no effect. On five-day-old biofilms, the range of CFU log reduction ± CI95% for all disinfectants and exposure times was of 0.28–3 (Table 2). Virocid still gave the highest reduction, whereas Virkon S’s reduction had fallen to the same level as TP990. There was a noticeable tendency of enhanced reduction for strains with BDAR morphology on two-day-old biofilm. However, the difference was only statistically significant using Virkon S and TP990 in the shortest exposure time on two-day-old biofilm (Figure 2). When applying Virkon S for 10 min. the effect was statistically different (*p* = 0.05) from that of using Virocid for 30 min. Further, the effect of TP990 for 15 min. and Virkon S for 10 min. were close to significantly different (*p* = 0.06).

## 4. Discussion

When testing three commonly used disinfectants on QREC strains from the broiler production chain in biofilm, we found the efficacy to primarily depend on biofilm age, exposure time, and disinfectant composition. The composition seemed to be the most important factor. Whereas all three disinfectants had a statistically significant reduction of biofilm, only two of them were able to meet the log_10_ CFU reduction requirement given in the NS-EN 13697:2015 under at least some of the conditions tested. The third disinfectant, TP990, was not sufficiently effective under any of the conditions.

The mode of action of the three disinfectants are different, but they are all combination products with different bactericidal/bacteriostatic agents. Virkon S is an oxidizing agent with an anionic surfactant and a low pH. The oxidizing agent in Virkon S is potassium peroxymonosulphate and its antibacterial action is suggested to be that it acts on bacteria by oxidation and on viruses by attacking the protein capsid [26,27]. For this reason, it is likely that the substance is effective against both the proteins in the matrix and the bacterial cell. Virocid is a quaternary ammonium compound (QAC) and a cationic surfactant in combination with glutaraldehyde and isopropanol. QACs are known to react with the cell membrane lipid bilayer while glutaraldehyde reacts with amines and thiol groups that are functional groups in proteins [28,29]. This can facilitate the penetration of biofilms as proteins being a component of biofilms matrices. TP990 is a diamine with acetic acid and an amphoteric surfactant, behaving as an anionic surfactant together with acids such as acetic acid. Previous studies have shown diamines to act directly on cell membranes and thereby also being effective on stationary cells [30,31]. The diamine used in TP990 is *N’*-(3-aminopropyl)-*N’*-dodecylpropane-1, 3-diamine is a fairly large molecule and to our knowledge not known to work on proteins. Nonetheless, the antimicrobial action has been demonstrated and the disinfectant as such has been shown to be active in planktonic studies. It is therefore possible that the size of the molecule and that it does not have a specific biofilm matrix disrupting agent may contribute to low penetration into the biofilm. However, relating the mode of action of disinfectants to effect on biofilm is an area that is little studied and needs to be further pursued in other studies.

The age of the QREC biofilm also proved to be important. Both Virocid and Vircon S had decreased bactericidal effect on five-day-old biofilms compared to two-day-old biofilms. Similar results have been reported when testing trisodium phosphate on 48- and 72-h old biofilms of *Salmonella enteritidis* on glass [32], as well as a quaternary ammonium compound and an enzymatic compound on five different *Salmonella* serovars in three and four day old biofilms on galvanised steel [33]. In the latter study, it was suggested that this may have been due to an increase in both biofilm thickness and the number of CFU/cm^2^ in the biofilm which was observed over time. In our study, the CFU was slightly increased in the untreated five-day-old biofilm compared to the two-day-old. It is therefore likely that the penetration of the disinfectant was reduced and incomplete in the biofilm formed for five days. It may therefore be necessary to increase the concentration and the contact time of the disinfectant compared to what the supplier recommends to account for changes occurring as biofilm matures. The above results also emphasize the importance of regular cleaning to avoid biofilm build up, as well as physical removal of biofilm before disinfection.

Exposure time could also be of importance. While Virkon S showed a satisfactory antimicrobial effect on the two-day-old biofilms after 30 min., this effect was significantly lower when the exposure time was reduced to 10 min. On the other hand, different exposure times for TP990 did not change the effect, supporting the hypothesis that TP990 does not act on the matrix and only affect bacteria in the outer layers of the biofilm. The exposure times used in this study were intended to be those recommended by the manufacturers. However, the suggested exposure times were given as a range, and there were variations in the proposed times. Due to this discrepancy, we chose to apply all disinfectants for 30 min., which was within the recommended range for all three, and in addition, 10 min. for Virkon S and 15 min for TP990. Our results show that increasing exposure times is beneficial when combatting QREC in biofilm, at least concerning certain disinfectants. This complies with other studies, which also found increased efficacy of various disinfectants with longer contact times [34,35,36,37].

In *E. coli,* curli fimbriae and cellulose determine the complex macroscopic architecture of the biofilm matrix and in RDAR, the most commonly found morphotype, both are expressed [5,13]. In our study, BDAR morphotype displayed a greater log_10_ CFU reduction than the RDAR and PDAR strains after treatment with all three disinfectants on two-day-old biofilm but not on five-day-old biofilm. This may suggest that cellulose is perchance important in withstanding disinfectants, at least in young biofilms but that other mechanisms of protection are more important in older ones. A protective effect of cellulose has been seen with chlorine [38] and also harmonizes with previous suggestions that cellulose can withstand strong acids and alkaline [12]. In addition, Virocid and Virkon S both contain active ingredients that directly affect proteins and not cellulose. This may be the reason that we found the effect of these disinfectants to be enhanced on BDAR, followed by PDAR and finally has the least effect on RDAR biofilms. Nonetheless, we could not make a firm conclusion on the variations in effect of the disinfectants on different morphotypes based on this study.

We also looked into the amount of CFU in the different morphotypes on untreated biofilm after two and five days and found little variation between these. However, the difference in CFU count between day two and five of strains with PDAR morphology was less than that of those with BDAR giving the impression that PDAR formed more biofilm in the initial phase of biofilm production. Of the two RDAR strains included in the study, one of the strains showed a similar pattern to the PDAR strains and the other to the BDAR strains. Yet, to conclude if this is a common tendency more studies are needed, especially since earlier studies show incongruent results concerning the role of cellulose and curli in biofilm. A synergy is suggested and it is proposed that cellulose might have a role in adherence [15]. Other studies performed on a *Salmonella* biofilm are suggesting that curli may be important in cell aggregation and cellulose in attachment to a surface [15,16].

Vast amounts of disinfectants are used globally today, and the surface disinfectant market alone has been estimated to USD 3.1 billion [39]. This will pose a potential risk on the environment and also a risk of the development of resistance in biofilm. Biofilm resistance is most often concerned with tolerance but stable resistance may also appear [40] and several mechanisms of resistance to disinfectants have been identified within biofilms [41,42]. The above concerns make it crucial to look towards novel and “greener” ways of microbial control. Several compounds have been identified as potent inhibitors of biofilm formation including substances obtained from nature [43,44]. Bridier et al. discussed, in a review from 2011, the importance of natural sources as a strategy in the management of biofilm. It is shown that certain essential oils may even be effective in eradicating established biofilms to levels close to that of a chemical disinfectants. [40,45,46]. Other studies have similar findings of effective essential oils on gram negative bacteria. One showed cinnamon to be especially useful on biofilm of gram negative organisms [47] and another found *L. domatiophorus* essential oil to show promising antimicrobial properties with a Minimum inhibitory concentration (MIC) and Minimum lethal concentration (MLC) of 2–8% *v/v* on *E. coli* [48]. Another fascinating future prospect discussed in Bridier et al.’s review was that natural and chemical agents may work in synergy and in this way be a powerful future substitute to other measures, such as increasing the dose of a chemical disinfectant [40,49,50]. Finding new solutions, which are neither harmful to the environment, animals, nor humans is an essential step in the future control of biofilm.

## 5. Conclusions

This study revealed differences between disinfectants in their ability to combat QREC in biofilm when following recommended user concentrations and exposure times. Furthermore, none of the disinfectants tested showed satisfactory effect against QREC in five-day-old biofilms. Even though our study was performed under optimal conditions and does not fully reflect a native environment, it does suggest a need for improving disinfectant susceptibility tests to include biofilms of different ages to ensure more real-world conditions. In addition, this study shows that there is a need for standard susceptibility tests to be developed on *E. coli* biofilms and not only on *Pseudomonas* biofilm as is the case today. Establishment of disinfectant routines (concentration, exposure times, frequency of treatment) should bear in mind these findings to prevent biofilm build-up that may be too laboursome to remove.

## Figures and Tables

**Table 1:** Strains, morphotype, and the mean log_10_ colony forming units (CFU) from biofilm after two and five days.

Strain	Morphotype	Mean ± SD from 2 Day Old Biofilm ^4^	Mean ± SD from 5 Day Old Biofilm ^4^
2914-01-7046-1	BDAR ^1^	7.02 ± 0.11	7.88 ± 0.52
2014-01-2069-1	BDAR ^1^	7.15 ± 0.19	8.12 ± 0.45
2014-01-5914-1	PDAR ^2^	7.91 ± 0.05	8.17 ± 0.32
2014-01-7342-1	PDAR ^2^	7.85 ± 0.27	8.08 ± 0.38
2014-01-2363	RDAR ^3^	6.40 ± 0.42	7.62 ± 0.72
2014-01-6040	RDAR ^3^	7.81 ± 0.21	8.20 ± 0.28

^1^ BDAR (Brown, dry and rough); ^2^ PDAR (Pink, dry and rough); ^3^ RDAR (red, dry, and rough); ^4^ Mean log_10_ CFU; SD = standard deviation.

**Table 2:** The disinfectants included in the study, active ingredients, the concentration applied, the treatment time and the mean log_10_ reduction for 2 and 5-day old biofilm.

Generic Name	Virkon S		Virocid	TP990	
Supplier	Lilleborg, Oslo, Norway		Agronor, Askim, Norway	Ecolab, Oslo, Norway	
**Group of active ingredients**	Oxidizing agent, maleic acid Sodium salts		Quaternary ammonium compound, cationic surfactant, aldehyde, and alcohol	Diamine with acetic acid amphoteric surfactant	
**Active ingredients**	Potassium peroxymonosulfate (40–55%) Malic acid (7–10%) Sodium chloride (10–12%)		Alkyl Dimethyl benzyl ammonium chloride (15–30%) Didectyl dimethyl ammonium chloride (15%) Gluteraldehyde (5–15%) Isopropanol (5–15%)	*N′*-(3-aminopropyl)-*N′*-dodecylpropane-1,3-diamine (3–5%) Acetic acid (1–2%) (*N*,*N*-Dimethylaminopropyl)trimethoxysilane (3–5%)	
**pH**	2.2–2.6		6.5	7.4–8.4	
**Concentration ^1^ (%)**	1		0.25	1	
**Exposure time (minutes)**	10	30	30	15	30
**Mean log_10_ red. 2- day ± CI95% ^2^**	1.71 ^A^ 0.87–2.55	3.34 ^B^ 2.05–4.63	3.23 ^B^ 1.71–4.75	0.95 ^C^ 0.56–1.34	1.08 ^C^ 0.42–1.74
**Mean log_10_ red. 5- day ± CI95% ^2^**	1.02 ^C^ 0.79–1.25	1.11 ^C^ 0.69–1.53	2.03 ^A^ 1.06–3	0.89 ^C^ 0.28–1.5	1.07 ^C^ 0.66–1.48

^1^ User concentration recommended by the manufactures; ^2^ log_10_ reduction and Confidence interval 95% (CI95%) on biofilm formed for 2 and 5 days. ^A, B, C^ Means with same letter are not statistically different (*p* > 0.05).
